# Effects of Low-Intensity Pulsed Ultrasound-Induced Blood–Brain Barrier Opening in P301S Mice Modeling Alzheimer’s Disease Tauopathies

**DOI:** 10.3390/ijms241512411

**Published:** 2023-08-03

**Authors:** Amandine Géraudie, Maximilien Riche, Thaïs Lestra, Alexandre Trotier, Léo Dupuis, Bertrand Mathon, Alexandre Carpentier, Benoît Delatour

**Affiliations:** 1 Paris Brain Institute, ICM, Inserm U1127, CNRS UMR 7225, Sorbonne University, 75013 Paris, France; maximilien.riche@aphp.fr (M.R.); lestrathais@gmail.com (T.L.); alexandre.trotier@icm-institute.org (A.T.); leo.dupuis@cea.fr (L.D.); bertrand.mathon@aphp.fr (B.M.); benoit.delatour@icm-institute.org (B.D.); 2 Department of Neurosurgery, Sorbonne University, APHP, La Pitié-Salpêtrière Hospital, 75013 Paris, France; alexandre.carpentier@aphp.fr; 3 Faculty of Medicine, Sorbonne University, GRC 23, Brain Machine Interface, APHP, La Pitié-Salpêtrière Hospital, 75013 Paris, France; 4 Advanced Surgical Research Technology Lab, Sorbonne University, 75013 Paris, France; 5 Laboratoire Des Maladies Neurodégénératives, Université Paris-Saclay, CEA, CNRS, 18 Route du Panorama, 92265 Fontenay-Aux-Roses, France; 6 Commissariat à l’Energie Atomique et aux Énergies Alternatives (CEA), Direction de la Recherche Fondamentale (DRF), Institut François Jacob, MIRCen, 18 Route du Panorama, 92265 Fontenay-aux-Roses, France

**Keywords:** Alzheimer’s disease, tauopathies, BBB opening, low-intensity pulsed ultrasounds

## Abstract

Alzheimer’s disease (AD) is the leading cause of dementia. No treatments have led to clinically meaningful impacts. A major obstacle for peripherally administered therapeutics targeting the central nervous system is related to the blood–brain barrier (BBB). Ultrasounds associated with microbubbles have been shown to transiently and safely open the BBB. In AD mouse models, the sole BBB opening with no adjunct drugs may be sufficient to reduce lesions and mitigate cognitive decline. However, these therapeutic effects are for now mainly assessed in preclinical mouse models of amyloidosis and remain less documented in tau lesions. The aim of the present study was therefore to evaluate the effects of repeated BBB opening using low-intensity pulsed ultrasounds (LIPU) in tau transgenic P301S mice with two main readouts: tau-positive lesions and microglial cells. Our results show that LIPU-induced BBB opening does not decrease tau pathology and may even potentiate the accumulation of pathological tau in selected brain regions. In addition, LIPU-BBB opening in P301S mice strongly reduced microglia densities in brain parenchyma, suggesting an anti-inflammatory action. These results provide a baseline for future studies using LIPU-BBB opening, such as adjunct drug therapies, in animal models and in AD patients.

## 1. Introduction

Alzheimer’s disease (AD) and other tauopathies are common and progressive neurodegenerative disorders that affect millions of people worldwide. The global prevalence of dementia was estimated to be 46.8 million in 2015, and it is projected to rise to 131.5 million by 2050 [1]. AD is the leading cause of dementia in older adults, while other tauopathies also count among the most frequent causes of dementia.

AD is neuropathologically characterized by extracellular deposits, mainly made of Aß peptides, resulting in the so-called amyloid plaques, and by intraneuronal neurofibrillary tangles arising from the aggregation of abnormally phosphorylated tau proteins. Despite large research efforts devoted to the study of AD physiopathology, there is no cure or effective treatment currently available for this devastating disease. Recently, passive anti-Aß immunotherapy trials and the FDA approval of two monoclonal antibodies have provided new hope [2]. These therapies, while positively acting on Aß deposit clearance, remain limited in their clinical efficacy, with a benefit-to-risk ratio still to be considered [3,4,5]. Due to a closer correlation between the burden of cortical tau aggregates and cognitive decline [6], anti-tau strategies could theoretically be more efficient than therapies tackling amyloid. Although drug development against tau assemblies was recently initiated [7,8,9], it has only demonstrated positive signs on biomarkers and negative results at a clinical level to date [8,9].

One key factor that may participate to AD therapeutic failure is the limited brain bioavailability of peripherally administered drugs. The blood–brain barrier (BBB), involving endothelial cells strongly interconnected by tight junctions, acts as a protective element to avoid penetration of toxins and pathogens in the central nervous system as well as to regulate the exchanges between brain and blood. The downside of the BBB is its limited allowance for blood-to-brain crossing of drugs, such as immunoglobulins with a brain concentration estimated to <0.1% of the peripheral dosage [10,11]. Some approaches have been developed to bypass the BBB barrier but are either invasive (e.g., direct intracerebroventricular injections) or associated with putative adverse effects (e.g., brain shuttles to hijack receptor-mediated transcytosis or intra-arterial hyperosmotic solutions to cause endothelial cell shrinkage) [11].

The use of ultrasounds to transiently open the BBB was discovered by Hynynen and colleagues [12]. Ultrasounds co-administered with intravenously injected microbubbles cause the latter to contract and expand rapidly, a process known as cavitation. This induces mechanical stress on the surrounding blood vessels, which leads to the loosening of tight junctions as well as increased transcytosis and fenestration, causing subsequent BBB permeation [13,14]. The safety of the technique has been repeatedly assessed, and numerous clinical applications have been approved by health authorities worldwide. In particular, BBB opening using ultrasounds was applied for the treatment of brain tumors through “enhanced chemotherapy” protocols by our group and others [15,16,17]. Different research teams (including ours) have also recently started to develop applications for AD patients [18,19,20].

In animal models of AD pathology, the use of focused ultrasounds with microbubbles (FUS) potentiates the action of therapeutic antibodies [21,22,23,24]. Surprisingly, a large number of preclinical studies also reported unexcepted beneficial effects of ultrasound-mediated BBB opening alone, without any adjunct drugs. Hence, in mice modeling Aß brain deposition, transient BBB opening mitigates amyloid burden [22,25,26], counteracts cognitive impairments [26,27], and, at the same time, potentiates resident immune cell activity [25,26,28,29] and stimulates neurogenesis [27]. Although relying on a limited number of publications, similar therapeutic actions of ultrasound-induced BBB opening were reported in mouse models of AD tauopathies [28,29,30].

The aim of the present study was to further characterize the effects of ultrasound-induced transient BBB opening in a transgenic mouse model developing tau pathology using low-intensity pulsed ultrasound (LIPU). In contrast to focused ultrasounds, LIPU allows a more diffuse opening of the BBB without the need for neuroimaging (MRI) guidance. LIPU safety has been previously assessed [31], as has its efficacy to potentiate chemotherapy treatment in glioblastoma, in both preclinical animal models and patients [15,16,17,32]. LIPU safety was also recently assessed in clinical trials enrolling AD patients [20]. To our knowledge, the disease-modifier effects of LIPU-induced BBB opening have never been assessed in preclinical models of AD. In the present study, we therefore performed a series of repeated LIPU-BBB openings in tau transgenic mice and analyzed the induced modulations of brain tau lesions and of innate resident immune cells (microglia). Our results showed that LIPU impacts both tau pathology and microglial burden. Overall, the collected data can be considered a pre-requisite for the subsequent development of LIPU-based therapies (e.g., BBB opening combined with therapeutic antibodies) and help to further comprehend LIPU’s potential for future translational applications (i.e., clinical trials in AD).

## 2. Results

### 2.1. LIPU Efficiently and Reproducibly Opens the BBB

A total of 45 mice were used in experiments (25 LIPU-sonicated and 20 non-sonicated mice) (Table 1).

**Figure 1:**
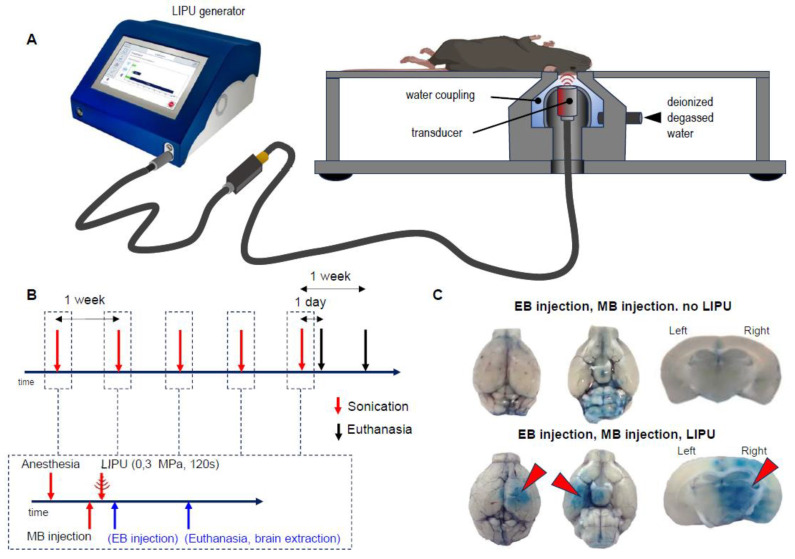
Experimental LIPU sonication setup. (**A**) preclinical platform used in mice. Ultrasounds are provided under the action of the generator, which transmits the required energy to the transducer, which vibrates at the determined frequency of 1 MHz. The transducer is placed in a chamber surrounded by degassed water. Mice are positioned on their backs on the platform, with their shaved skulls in direct contact with the deionized and degassed water. The mouse head is placed to expose the right hemisphere to the ultrasound beam. (**B**) processing of the whole procedure of sonication (upper panel) with a zoom on a sonication session (lower panel). The procedure for BBB opening assessment using Evans Blue is indicated in blue. (**C**) visualization of Evans Blue dye on whole brains (left and middle macrographs corresponding to dorsal and ventral views, respectively) and coronal blocks of brains (right macrographs) in non-sonicated (upper panels) and LIPU-sonicated (lower panels) mice. Extravasation of Evans Blue is evidenced in LIPU-sonicated brains in the right hemisphere that is targeted by LIPU (red arrowheads), while staining remains peripheral with no signs of parenchymal diffusion in non-sonicated brains. EB: Evans Blue, LIPU: low-intensity pulsed ultrasounds, MB: microbubble.

All mice, but two, survived repeated anesthesia and sonication procedures (intravenous injections, LIPU BBB opening, etc.; see Figure 1A,B), and no adverse events were observed during the experiment time course.

The efficiency and reproducibility of LIPU-induced BBB opening were controlled for all sessions by visual examination of Evans Blue brain extravasation in sonicated test mice (i.e., 1 mouse/sonication session) (Figure 1B,C).

In all sonicated test mice, whole brain and coronal tissue block observations revealed the presence of the blue dye in the right hemisphere of the brain, confirming the BBB opening (see Figure 1C, lower panel). In previous experiments, a series of mice received the same procedure but without the application of ultrasounds. Under these conditions, the mouse brain did not show any blue intra-parenchymal coloration, confirming that the BBB was intact (see Figure 1C, upper panel). These results claim the reproducibility and efficiency of BBB opening mediated by LIPU.

### 2.2. Lateralization Effect on the Levels of Immune-Staining

For all analyses, no difference was demonstrated between left and right hemispheres in sonicated mice, regardless of the immunostaining considered (AT8, Iba1, GFAP, see Figure S1 and Table 2), except in the amygdala for AT8 H-scores at 7 days post-sonication (however, group comparisons between LIPU-sonicated and non-sonicated mice led to the same results on AT8 H-scores in the amygdala when statistical analyses were performed on the whole brain or only on the right hemisphere). Thus, bilateral measures, collected in both the right and left hemispheres, were used for further analyses.

### 2.3. LIPU-Induced BBB Opening Does Not Decrease Tau Pathologies

To investigate the effect of LIPU on tau pathology development, we analyzed and compared tau-positive lesions between sonicated and non-sonicated P301S mice (Figure S2A–C).

**Figure 2:**
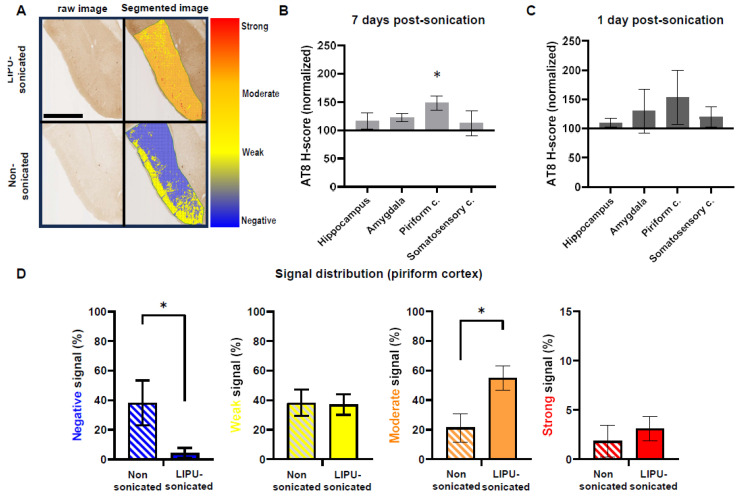
Effects of LIPU on tauopathies. (**A**) Tau pathology in raw images (left panels) and as quantified by H-score (right panels) in the piriform cortex of a non-sonicated mouse (upper panels) and a LIPU-sonicated mouse (lower panels) (scale bar = 1 mm). The overall increase in signal intensities and distribution in the LIPU-sonicated brain can be observed. (**B**) AT8 H-scores of LIPU-sonicated P301S mice (*n* = 10) normalized to H-scores of non-sonicated P301S mice (*n* = 6), seven days after the last sonication session. An overall increase in H-scores is observed in all brain regions of LIPU-sonicated mice, but statistical significance between groups is only reached in the piriform cortex. (**C**) AT8 H-scores of LIPU-sonicated P301S mice (*n* = 4) normalized to H-scores of non-sonicated P301S mice (*n* = 4), one day after the last sonication session. A trend for an overall increase in H-scores is observed in all brain regions of LIPU-sonicated mice. (**D**) Comparison and distributions of negative (blue), weak (yellow), moderate (orange), and strong (red) AT8 signal intensities in the piriform cortex of LIPU-sonicated (*n* = 10) and non-sonicated (*n* = 6) P301S mice. Compared to non-sonicated mice, LIPU-sonicated mice display an increase in moderate intensity signal and a concurrent drop in negative (background) signal, underling a global intensification and spreading of AT8 immunoreactivity after LIPU sonication (* *p* < 0.05).

In contradiction with our initial working hypothesis (based on previous observations in FUS-treated mice), results revealed that LIPU-induced BBB opening did not decrease tau pathology in the explored regions. Conversely, LIPU-mediated BBB opening was associated with increased tau pathology in specific ROIs, with a significant increase of 48% (*p* < 0.05) in the piriform cortex when compared to non-sonicated mice (Figure 2A,B). The analysis of the signal intensities distributions indicated that the increase in H-score in the piriform cortex was derived from a decrease in negative signal (background) and a concomitant increase in moderate signal, with no changes in high signal (Figure 2D). This suggests that repeated transient BBB openings favor, at least in the piriform cortex, the dissemination of early tau accumulations (presumably at pre-tangle stages), while more mature and aggregated forms of tau deposition (i.e., standard neurofibrillary tangles) were unaffected by treatment.

Since these results were not consistent with preceding studies depicting an ultrasound-induced reduction of tau burden, we wondered if there may be some methodological differences that could account for these discrepancies. We noticed that in previously published studies [28,29,30], mice were euthanized one to three days following the last session of sonication. We therefore suspected an effect of the post-sonication delay underlying dynamic changes of tau pathology over time after BBB opening (i.e., acute decrease of tau pathologies followed by normalization and even possible potentiation of the lesions at a later time). To test this hypothesis, we performed a new set of experiments by sacrificing mice one day (instead of seven days) after the last sonication. However, very similar results of LIPU-mediated BBB opening were observed at 1 day post-sonication (Figure 2C and Figure S3): no decrease of H-scores in sonicated mice but, on the contrary, an overall tendency for increased H-scores with a comparable region-dependent profile as observed at 7 days post-sonication. However, H-score increases in mice euthanized one day following the last sonication session did not reach significance in any specific regions, presumably due to a lack of statistical power (*n* = 4 mice in each group).

Taken together, these results suggest that LIPU-mediated BBB opening does not induce a decrease in tau pathology, as opposed to results reported in previously published studies, and may even cause an increase in tau pathology in selected brain regions. Additionally, these results did not seem to be influenced by the time delay (short or long) between the last session of sonication and euthanasia.

### 2.4. LIPU-Mediated BBB Opening Induces Genotype-Dependent Modulation of (Micro)Glial Cells

#### 2.4.1. Decreased Microglial Densities in P301S Tau Transgenic Mice

**Figure 3:**
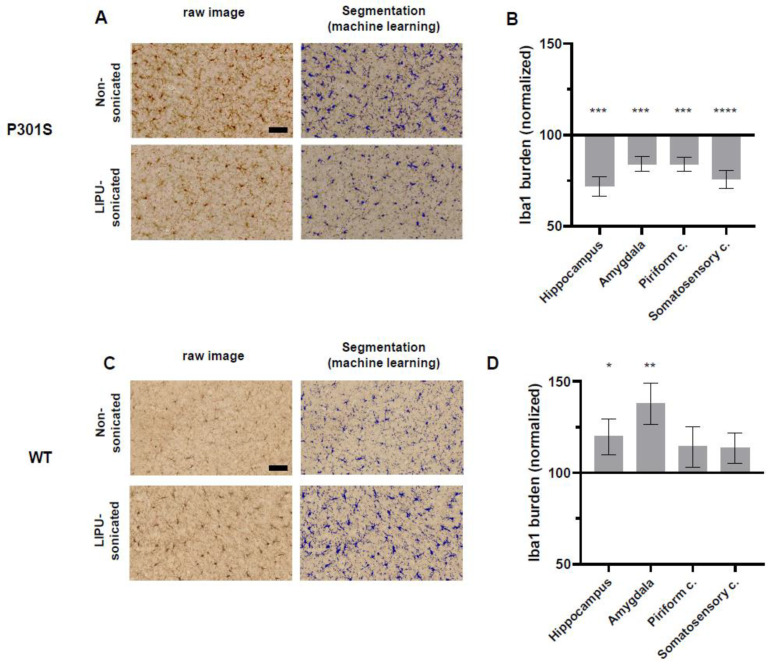
Effects of LIPU on microglial cells in P301S and WT mice. (**A**) Iba1-stained images in raw images (left panels) and as quantified by a machine-learning algorithm (right panels) in the somatosensory cortex of a non-sonicated (upper panels) and a LIPU-sonicated (lower panels) male P301S mouse (scale bar = 50 µm). Note the overall decrease in microglial burden in the LIPU-sonicated brain. (**B**) Iba1 loads of LIPU-sonicated P301S mice (*n* = 10) normalized to loads of non-sonicated P301S mice (*n* = 7). In all brain regions analyzed, a significantly reduced microglia burden is observed after sonication. (**C**) Iba1-stained images in raw images (left panels) and as quantified by machine-learning algorithms (right panels) in the somatosensory cortex of a non-sonicated (upper panels) and a LIPU-sonicated (lower panels) wild-type mouse (Scale bar = 50 µm). Note the overall increase in microglial burden in the LIPU-sonicated brain. (**D**) Iba1 loads of LIPU-sonicated WT mice (*n* = 8) normalized to loads of non-sonicated WT mice (*n* = 9). A general trend was observed in all brain regions for an increased microglia burden after sonication (a significant increase in the hippocampus and amygdala) (* *p* < 0.05; ** *p* < 0.01; *** *p* < 0.005; **** *p* < 0.0005).

We then evaluated the effects of LIPU-induced BBB opening on the microglial cell load in the different regions of interest using anti-Iba1 immunostaining (Figure S2D–F). Our results revealed a consistent decrease in microglial immunostaining in sonicated P301S mice in all explored regions. Specifically, a reduction of Iba1 load of 28% in the hippocampus (*p* < 0.005), 16% in the amygdala and piriform cortex (*p* < 0.005), and 24% in the somatosensory cortex (*p* < 0.0005) (Figure 3A,B).

To further investigate the origin of the LIPU-induced decrease in microglial burden, we quantified the number of Iba1-positive cell soma. Quantification of Iba1+ soma number indicated that LIPU BBB opening significantly decreased the number of microglia in the hippocampus (−22%, *p* < 0.05) and in the somatosensory cortex (−23%, *p* < 0.05), as well as exhibiting a trend towards a diminution in the microglia number in the piriform cortex (n.s; *p* = 0.07) of sonicated P301S mice in comparison to non-sonicated mice (Figure S4A). These results underline that the reduced Iba1 burden induced by LIPU can be explained by a decrease in microglial densities after treatment.

To evaluate the overall impact of LIPU-BBB opening on glial cells, we finally assessed astrocytic loads using GFAP staining. Our results did not reveal any statistically significant changes in GFAP immunostaining between sonicated and non-sonicated mice, suggesting comparable levels of astrocyte activation in the two groups (Figure S4B).

#### 2.4.2. Increased Microglial Loads in Wild-Type Mice

To further comprehend the effects of LIPU-mediated BBB opening on microglial cells, we evaluated the effects of sonication in wild-type mice. We used male and female WT mice and combined the results, as their baseline microglial load did not show any sexual dimorphism. Our results showed that, in sharp contrast with our observations in P301S transgenics, microglial loads were increased in wild-type mice after sonication, with a 38% (*p* < 0.01) increase in the amygdala and a 20% (*p* < 0.05) increase in the hippocampus when compared to non-sonicated WT mice (Figure 3C,D).

Altogether, these results show that LIPU-induced BBB opening may have a repressing effect on microglia in mice harboring tau lesions but also suggest that this effect might be disease-specific as it was not observed in healthy control mice.

## 3. Discussion

### 3.1. Summary of Results

Focused ultrasounds combined with microbubbles are an innovative and safe strategy to open the BBB and have been shown to promote beneficial effects on both AD brain pathologies as well as neurogenesis and cognitive decline. Our study assessed the effects of low-intensity pulsed ultrasounds, which provide more diffuse BBB opening than focused ultrasounds, on tau pathology and microglia in transgenic P301S mice that develop tau pathology. We first showed that the application of LIPU to P301S and WT mice is able to open the BBB safely, efficiently, and reproducibly. We then demonstrated that, contrary to previously published studies using focused ultrasounds, LIPU-sonicated mice did not display a decrease in tau pathology but instead showed a potentiation of phospho-tau accumulation, remaining modest in 3/4 of the brain regions analyzed and more prominent in the piriform cortex. In addition, LIPU induced a strong and widespread reduction of microglia densities in P301S mice (possibly reflecting an anti-inflammatory action) and, conversely, an increase in microglial loads in wild-type mice.

### 3.2. Efficiency and Safety of LIPU-Induced BBB Opening

Our results confirm BBB permeabilization under the action of LIPU associated with microbubbles. Indeed, half an hour to one hour after the application of LIPU, Evans Blue extravasation into brain tissue was visually detected in all test mice, while no parenchymal coloration was visualized in the non-sonicated hemisphere as well as in non-sonicated mice. Evans Blue does not cross the BBB in physiological conditions, meaning that the BBB was permeabilized in LIPU-sonicated mice. No side effects were observed in the mice that received the whole procedure (five sonication sessions). The two mice who died during the protocol underwent only one sonication, and it is therefore difficult to invoke the deleterious effects of repeated LIPU-BBB openings to explain these deaths. Our results are consistent with numerous previous publications in diverse animal models (and in humans) showing that LIPU can be applied repeatedly and safely to efficiently and reproducibly open the BBB [12,33,34].

### 3.3. Comparison with Previous Studies of Ultrasound-Induced BBB Opening in AD Mouse Models

Our results contrast with previous reports from the literature, and several factors may explain these discrepancies. Firstly, different mouse models were used across all previously published studies. Most studies exhibiting beneficial effects of ultrasound-mediated BBB opening in AD mouse models were conducted in amyloid mouse models [22,24,25,26,27,35]. Moreover, the effects of ultrasound-BBB opening on mice harboring tau lesions have been assessed in different models (rTg4510 [29] and K3 lines [28,30]), but to the best of our knowledge, not in the widely used P301S (PS19 line) transgenics used in our study. Importantly, the different tau transgenic models have various distributions, magnitudes, and kinetics of neuropathological lesions, including tau deposition and neuroinflammation. Therefore, the application of the BBB opening procedure at different stages of pathology and in various disease models may result in the specific and distinct effects observed and reported in the aforementioned studies. Critically, in our study, LIPU treatment started in 4.5 month old P301S mice, which are not supposed to already show mature tangles [36], whereas Karakatsani et al. [28] initiated their treatment at 3.5 months of age in rTg4510 mice, which already show pre-tangle-like structures [37]. Moreover, brain regions analyzed in these studies were either limited to the hippocampus or involved both the hippocampus and cortex, but none of them explored separately the piriform cortex, the amygdala, or the somatosensory cortex as in the present work [28,29,30]. Secondly, various types of ultrasounds have been used in studies assessing the effects of ultrasound-mediated BBB opening. Previous reports used focused ultrasounds [22,25,28], sometimes associated with a device allowing the entire scalp to be covered with multiple points, i.e., the scanning ultrasound technique [26,29,30]. In addition to their focused or diffuse nature, the parameters of ultrasounds also varied largely between research studies. Some protocols used a high center frequency (1.5 MHz [28]), others applied heavier peak acoustic pressure (0.45 MPa [28], 0.5 MPa [29], and 0.65 MPa [30]), or a pulse repetition frequency (10 Hz [29,30]) involving greater energy delivery to the brain. Our study used low-intensity pulsed ultrasounds, which allow a more diffuse BBB opening and have never been studied in AD animal models. This type of ultrasound has previously been used mainly in studies associating LIPU-mediated BBB opening and chemotherapy in the treatment of brain tumors, allowing BBB permeabilization as well as higher chemotherapy brain intake and greater overall survival [17,32]. The number of sonication sessions also differed largely (from 4 to 15) between studies. Another factor that may greatly impact the study outcomes is the delay between the last session of sonication and the ensuing brain extraction for neuropathological assessment. In previous studies in tau transgenic models, mice were euthanized with a shorter delay (from one to three days) than in our study [28,30]. By investigating the effects of three different delays before euthanasia (4 h, 4 days, and 15 days), Jordao et al. showed that the increase in microglial load appeared as soon as at 4 h, peaked at 4 days, but was not any more significant after 15 days [25]. In our study, the delay between the last session of sonication and euthanasia did not seem to impact the effects of LIPU-BBB opening on tau pathology; however, this should be further confirmed in a larger number of mice along with the assessment of the delay’s influence on the microglial population.

### 3.4. Hypotheses to Explain the Impact of LIPUs on Microglia and Tau

While several factors may explain the divergence of our results from those of the literature, we suggest some hypotheses to explain our main observations: a moderate increase in tau pathology and a decrease in microglial burden, as well as a possible link between these two effects of LIPU treatment. Our results showed a decrease in microglial loads in P301S mice, which was not reported in previous studies using AD mouse models [25,26,28,29]. This modulation of microglia seems to depend on the specific underlying pathological state of P301S mice. Indeed, LIPU-induced BBB opening increased microglial loads in wild-type mice. 

Previous studies have reported contradictory results concerning the effects of BBB opening with ultrasounds in wild-type mice. While some observed an increase in microglial levels and activation state [25,26], paralleling our results, others have shown a decrease in gliosis and microglial activation following sonication [38]. One study, using LIPU, did not report any change in microglial levels in the hippocampus of wild-type mice [31]. In the latter study, the background of mice (C57BL/6J) differed from the one used in our work (P301S mixed genetic background), as did the mice’s age (2 months vs. 7 months) and number of sonication sessions (one isolated session vs. 5 repeated sessions). Furthermore, only the hippocampus microglial burden was assessed, while we analyzed multiple regions of interest.

We showed that the effects of LIPU on microglia are disease-dependent, as opposite effects were observed in wild-type mice (increased load) and P301S transgenics (decreased load and cell numbers). P301S mice have the particularity of developing early microglial activation at 3 months of age [36]. This particular phenotype might be required for the specific taming effects of LIPU observed in the present study. Interestingly, we did not see any significant changes in astrocytic load in P301S mice, suggesting that the effects of ultrasound-mediated BBB opening on neuroinflammation may be complex and still misunderstood. In particular, the modulations of Iba1 immunoreactivity that we quantified need to be further characterized in both wild-type and tau transgenic P301S mice (e.g., assessment of microglia fine morphologies by skeleton analysis, additional histological and biochemical markers to document microglia states and transitions, etc.).

We may speculate that reduced Iba1 burden in sonicated P301S mice is the source of tau modulations. Indeed, it has been shown that microglial cells can internalize and phagocyte tau, participating in its clearance from the brain parenchyma [39]. Therefore, the decrease in microglial burden may impair the phagocytosis of tau and other aggregated debris and thus limit homeostatic tau clearance. However, we did not observe any negative correlation between Iba1 and AT8 metrics. This lack of association does not support the hypothesis that microglial tempering is linked to increased tauopathies. Furthermore, it has been described that microglia could paradoxically play a key role in tau pathology progression and spread through the brain [40,41]. According to these observations, the induced decrease in microglia observed in LIPU-sonicated P301S mice should theoretically have led to beneficial effects on tau pathology. However, this potential positive effect on tau pathology was clearly not observed in our study. We therefore favor the hypothesis that LIPU acts on both tau lesions and microglial cells and that these modulations are independent.

It remains possible that LIPU sonication may cause an increase in tau pathology by inducing conformational changes in protein assemblies and possibly a “disintegrating effect” of multimeric formations. New conformers or shorter assemblies may be able to accelerate seeding and promote spreading of misfolded forms of tau, leading to an overall increase in tau pathology in sonicated mice. Our signal intensities analysis showed a larger occupancy of moderately stained phosphorylated tau immunoreactivity after repeated LIPU treatments. It would be interesting to evaluate if increased levels of tau oligomeric forms could be detected in parallel to these histological findings. The specific changes in these tau assemblies cannot be captured with the AT8 antibody we used. Tau oligomeric forms possibly increased by LIPU-BBB opening could be assessed using both specific antibodies (e.g., T22 antibody) and/or biochemical dosages. Higher-order aggregates (i.e., mature tangles) could also be evaluated using dedicated antibodies (e.g., AT100, AT180). This will extend our understanding of the effects of LIPU-induced BBB opening in tau pathology and, in particular, refine the contribution of high-order aggregates versus oligomeric species. Moreover, it could be of great interest to evaluate the potential effects of LIPU-induced BBB opening on the propagation of tau pathology using standard inoculation paradigms [42].

### 3.5. Limits

A limitation to our study is the relatively low number of mice used in each experiment, particularly when looking at the effects of delay between LIPU’s last sonication session and euthanasia. Furthermore, it is well known that P301S mice display a high degree of variability in their pathology [43], which also limits the statistical power of the analyses. Larger groups of mice would be needed to firmly conclude on the effects of LIPU.

## 4. Materials and Methods

### 4.1. Animals

P301S tau transgenic mice (PS19 line #008169, Jackson Laboratory, Bar Harbor, ME, USA) were used as well as wild-type (WT) littermates. P301S tau transgenic mice are established on a mixed C57BL/6 x C3H background and express the mutant P301S form of the microtubule-associated protein tau (MAPT) human gene under the control of the mouse prion protein promoter. This transgene encodes the P301S pathogenic mutation and includes 4 repetition domains and an N-terminal insert (4R/1N), leading to a 5-fold over-expression of human tau proteins [36]. Tau transgenic P301S mice develop accelerated tau propagation as early as age 1.5 months [44], but also microglial and astrocytic activation and synaptic impairment as early as age 3 months [36]. Classical neurofibrillary tangle-like inclusions appear around 5–6 months of age [36]. Of note, it has been described that male P301S mice display more extensive tauopathies than their female counterparts [45]. The present study focused on male transgenic mice to minimize confounding factors. Concerning WT mice, both males and females were used, as no sex differences were observed in this genotype for the collected variables and used metrics.

All the mice were housed in individual ventilated cages at the ICM’s pathogen-free animal facility. Transgenic mice were co-housed with their WT littermates; bedding material and wood chips were available in in-home cages as enrichment. The animals were maintained in a controlled environment with ad libitum access to food and water, a constant temperature (19–22 °C), and humidity (40–50%) on a 12/12 h lightness-darkness cycle regime.

The number of mice and the experimental conditions used for each experiment in this study are summarized in Table 1. Mice were randomly allocated to experimental groups.

All experimental studies were performed in accordance with the European Committee Council Directive (2010/63/UE), followed ARRIVE guidelines (https://arriveguidelines.org/; URL accessed on 8 January 2023) and were approved by the local ethics committee and the French ‘Ministère de l’Enseignement Supérieur, de la Recherche et de l’Innovation’ (APAFIS N°22117-201909201708860).

### 4.2. Low-Intensity Pulsed Ultrasound BBB Opening Procedure

BBB opening was obtained using low-intensity pulsed ultrasound (LIPU), associated with intravenous injection of microbubbles. LIPU was performed using the LIPU pre-clinical system (SonoCloud Technology, CarThera, Paris, France), which consists of a 10 mm-diameter ultrasound transducer surrounded by a cylinder filled with previously degassed and deionized water to facilitate acoustic wave transmission and coupled to the head of the mice at a 15 mm distance from the transducer (Figure 1A). The parameters were applied as follows: the center frequency was 1 MHz using a pulse length of 25,000 cycles with a pulse-repetition frequency of 1 Hz and an acoustic pressure of 0.3 MPa, as measured in water, for a total duration of 120 s. These parameters were previously optimized and defined as safe in mice [12,33,34]. We will subsequently refer to this procedure as the sonication procedure.

The complete LIPU sonication procedure was performed as follows. Mice were anesthetized using an intraperitoneal injection of Ketamine (100 mg/kg, solution of 100 mg/mL, Imalgène©, Virbac, France) and xylazine (10 mg/kg, Rompun© 2%, Bayer, La Garenne-Colombes, France). The head was shaved and depilated, and xylocaine (tetracaine 0.4%) was applied in each eye for local analgesia. After 2 min, 100 to 120 µL of microbubbles (Echography contrast SonoVue©, Bracco Imaging, Évry-Courcouronnes, France) were intravenously injected in the retro-orbital sinus after their reconstitution with physiological serum. A few seconds after the injection, mice were positioned on the ultrasound platform on their backs with their heads maintained in contact with the water and lateralized on their right to target the right hemisphere with the ultrasound beam. LIPU was applied as described above. Non-sonicated (control) mice followed the same protocol except that the ultrasounds were not applied once the mice were placed on the sonication platform. Throughout the whole procedure, except for the time spent for intravenous injection and on the sonication platform, mice were kept in a thermoregulated cage (38 °C) until awakening.

A sonication session was performed every week for 5 weeks (Figure 1B). Given the experimental group, mice were sacrificed one day or seven days after the last session of sonication by intraperitoneal injection of pentobarbital (Euthasol©, Cerba Vet, Massy, France, 120 mg/kg). Mice were then intracardially perfused with 4% paraformaldehyde. Brains were extracted and post-fixed for 24 h in the same solution.

### 4.3. BBB Opening Assessment

To verify the efficacy and reproducibility of the LIPU-mediated BBB opening for each sonication session, one extra-mouse (transgenic or WT mouse, not allocated to experimental groups) was intravenously injected with Evans Blue (6% in physiological serum) just after the application of the ultrasounds (Figure 1B). It is known that Evans Blue binds to albumin once intravascularly injected and therefore forms a high-molecular-weight macromolecular complex that does not cross the BBB and remains in peripheral blood vessels in physiological conditions but is able to diffuse in brain parenchyma when the BBB is permeabilized by sonication (Figure 1C). For these control assays, mice were sacrificed 30 min to 1 h after dye injection, and the blue staining of the brain parenchyma was visually and macroscopically inspected after brain extraction and dissection.

### 4.4. Mouse Brain Tissue Preparation

Fixed brains were cryoprotected in a dimethylsulfoxide (2%)—glycerol (20%) solution for 24 h. After cryoprotection, brains were cut using a sliding freezing microtome into 40 µm-thick free-floating coronal sections that were stored at −20 °C in the cryoprotectant. In order to identify the sonicated (right) hemisphere, a hole was made into the non-sonicated (left) hemisphere using a small-diameter needle (27G). Serial sections were collected from the rostral (frontal pole) to the caudal (brainstem, cerebellum) parts of each brain.

### 4.5. Immunohistochemistry

Endogenous peroxidases were inactivated using hydrogen peroxide (3% H_2_O_2_, 40% methanol) for a 10 min incubation. Free-floating sections were then incubated for an hour in a PBS triton solution (0.25%) supplemented with bovine serum albumin (4%), to minimize non-specific binding. Brain sections were then incubated in a primary antibody solution overnight at room temperature: biotinylated AT8 (p202/p205, Thermo Fisher, Les Ulis, France, 1:1000, MN01020B), anti-ionized calcium binding adaptor molecule—Iba1 (Wako-Thermo Fisher, les Ulis, France, 1:1000, 019-19741), or anti-glial fibrillary acidic protein—GFAP (Abcam, Paris, France 1:5000, AB7260). The next day, all slices (with the exception of sections incubated with primary biotinylated AT8) were incubated in biotinylated secondary antibodies for an hour and a half at room temperature (goat anti-rabbit, Vector laboratories—Eurobio Scientific, Les Ulis, France, 1:250, BA-1000). Biotin residues were finally detected using the avidin-biotin-peroxidase technique and revealed by the oxidation of diaminobenzidine (DAB, Sigma Aldrich, Saint-Quentin-Fallavier, France). Free-floating slices were then mounted on SuperFrost slides, dehydrated, and cleaned in alcohol and xylene baths. Eukitt© Mouting Medium (Electron Microscopy Sciences—CliniSciences, Nanterre, France) was used to mount the slices. The obtained slices were then scanned using a Nanozoomer scanner (Hamamatsu Photonics, Massy, France) at a 40× magnification.

### 4.6. Microscopic Morphological Analysis

The obtained scanned images were analyzed with QuPath ([46], version 0.4.3) and ImageJ (National Institutes of Health, Bethesda, MD, USA) software. An average of four slices per animal were chosen at anatomical levels, including all regions of interest (ROI). Four ROIs, known to harbor tauopathies in P301S transgenics, were manually outlined from these brain slices: the hippocampus, amygdala, piriform, and somatosensory cortex. 

AT8 staining was quantified following these sequential steps (Figure S2A–C):

(1) Segmentation of all ROI in superpixels, which adapt their shape to local staining contrasts instead of using a rigid and regularly spaced grid. We made use of the 10 µm QuPath tool ‘SLIC superpixels’;
(2) Intensity analysis of each superpixel was classified on the basis of thresholds remaining constant across images of the same experience, including sonicated and non-sonicated mice: no signal (negative), weak signal, moderate signal, strong signal;
(3) Calculation of an H-score [47] as follows:

$$H-score=\frac{{SP}_{w}\times 100+{SP}_{M}\times 200+{SP}_{H}\times 300}{{SP}_{T}},$$

SP_W_:  number of superpixels with weak signals;
SP_M_:  number of superpixels with moderate signals;
SP_H_:  number of superpixels with high signals;
SP_T_:  number of superpixels (total).

The obtained H-score varies from 0 to 300 and allows for a dynamic range in estimating staining levels (including both surface area stained and intensity of labeling). For each mouse, the proportion of each intensity category (null, weak, moderate, and strong) was also extracted. H-scores were assessed in the present study in order to take into account not only the signal of densely stained mature neurofibrillary tangles but also the presence of pre-tangles as well as more diffuse staining. Previous attempts to analyze discrete tau deposits varying in nature and topography have been made [48], but using a qualitative approach. H-scoring allows one to quantitatively assess pTau-immunostained material.

Iba1 staining was analyzed in each ROI using a machine-learning algorithm in QuPath (Figure S2D–F). The algorithm was trained with several images with positive (microglial staining) and negative (background) manual annotations of different representative regions. Once trained, the model was able to efficiently recognize Iba1 staining and differentiate it from background noise. Comparing machine-learning segmentation vs. standard and conventional (thresholding) methods clearly underlined the better performances of the former. We extracted the percentage of stained area reported from the total area in order to calculate the overall Iba1 load.

To quantify the number of Iba1 microglial cell soma and calculate cell densities, we used an ImageJ macro command: (1) eight-bit conversion; (2) local contrast normalization; (3) auto-thresholding; (4) closing; (5) erosion; (6) despeckle; and (7) particle counting (size cut-off: 8 µm^2^–infinity). Finally, counted particles were normalized by the ROI total area to provide cell densities in each ROI.

GFAP staining was assessed using Phansalkar thresholding (adapted to finely branched objects such as astrocytes) on ImageJ to extract the stained area in each ROI. The ‘stained area/total area’ ratio allowed for the extraction of the percentage of immunostained area (i.e., GFAP load).

All the image analyses were performed blindly for the experimental groups (LIPU-sonicated or non-sonicated).

### 4.7. Statistical Analyses

Statistical analyses were performed using Prism (version 9.4.1) software (Graphpad, San Diego, CA, USA). Of note, one animal was excluded from the analysis of AT8 H-scores (a non-sonicated mouse) as the metrics differed significantly (outlier test) in this mouse vs. other mice in the same group. Additionally, one wild-type mouse was excluded from analysis due to numerous tissue artefacts. Nonparametric paired Wilcoxon tests were performed to assess possible inter-hemispheric differences (left vs. right). As no difference between hemi-brains was observed (see Section 2.2), bilateral measures were used for further analyses. Pairwise comparisons were performed using nonparametric Mann–Whitney tests to compare sonicated vs. non-sonicated animals. A *p*-value was considered statistically significant when <0.05 (*: *p* < 0.05; **: *p* < 0.01; ***: *p* < 0.005; ****: *p* < 0.0005). For the sake of clarity in the figures and to be able to compare sonication effects (i.e., % decrease/increase) in various brain ROIs that display large variations in baseline immunoreactivity levels, our results are represented as normalized to the mean of non-sonicated animals of each experience (mean of non-sonicated mice measures in one experience set at 100%). All data are represented with the mean ± SEM in graphs.

## 5. Conclusions

Taken together, our results indicate that LIPU safely provides an efficient and reproducible opening of the BBB. Additionally, this work suggests that, in P301S mice, LIPU-induced BBB opening alone, without any adjunction of drug, does not decrease tau pathology and that this lack of effect is observed at both short and longer delays following sonication. In parallel, BBB opening using LIPU in tau transgenic mice promotes a clear reduction of the microglia population number, an “anti-inflammatory” effect that seems to be disease-specific and not observed in normal mice.

Our findings provide a baseline on which future preclinical trials using LIPU could be further developed, such as BBB transient opening to improve the penetration of drugs into brain parenchyma and therefore maximize bioavailability at target sites. Finally, our results also bring new information on this innovative approach that may be translationally applied in the future to patients with AD.

## Figures and Tables

**Table 1:** Mice groups.

Genotype	Sex	Experimental Group	Delay between Last Sonication and Euthanasia (Days)	Mean Age at Euthanasia (Months)	*n*
P301S	Male	LIPU-sonicated	7	5.7	10
		Non sonicated	7	5.9	7
	Male	LIPU-sonicated	1	6.5	5
		Non sonicated	1	6.5	4
WT	Male	LIPU-sonicated	7	6.7	5
		Non sonicated	7	6.7	4
	Female	LIPU-sonicated	7	6.8	5
		Non sonicated	7	6.7	5

**Table 2:** Comparison of left and right hemisphere immunostaining levels for all studied markers.

Region of Interest	AT8-Day 7	AT8-Day 1	Iba1	GFAP
Hippocampus	ns	ns	ns	ns
Amygdala	*p* < 0.05	ns	ns	ns
Piriform cx	ns	ns	ns	ns
Somatosensory cx	ns	ns	ns	ns

## Data Availability

The raw data presented in this study are available on request from the corresponding author.

## Supplementary Materials

The following supporting information can be downloaded at: https://www.mdpi.com/article/10.3390/ijms241512411/s1.
