# Supplementary material for: Effects of Low-Intensity Pulsed Ultrasound-Induced Blood–Brain Barrier Opening in P301S Mice Modeling Alzheimer’s Disease Tauopathies
Source: Int J Mol Sci. 2023 Aug 3;24(15):12411. doi: 10.3390/ijms241512411 (PMC10419069; doi:10.3390/ijms241512411)
Supplement: Supplementary file 1 [file ijms-24-12411-s001.zip › ijms-2505839-supplementary.pdf]

**Supplementary material**

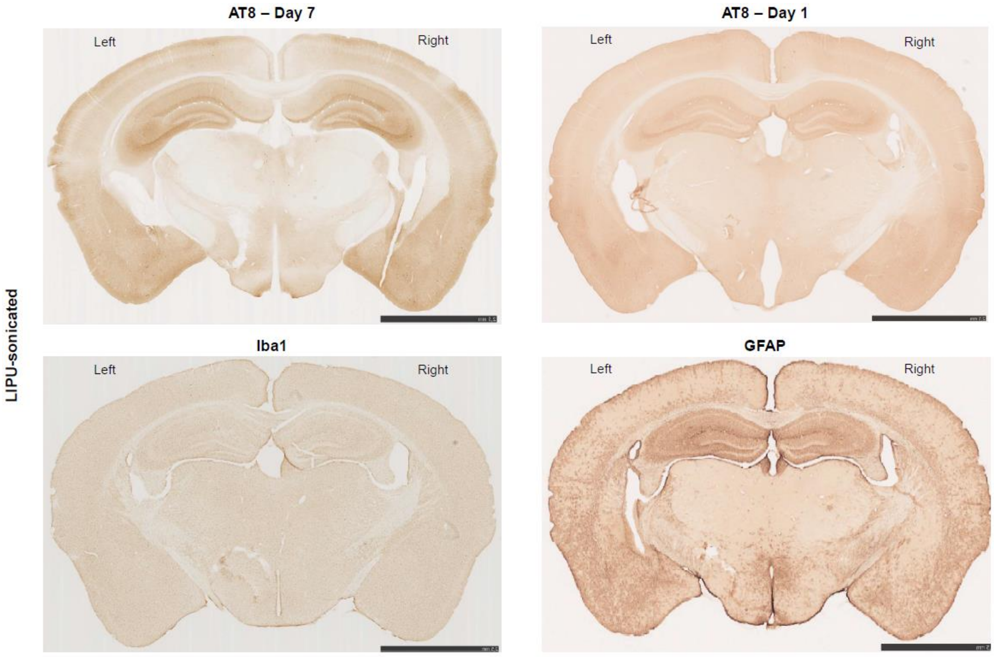


**Figure S1.** Brain slices immuno-stained with AT8 (from brains harvested 7 days or 1 day after the last session of sonication), anti-Iba1 and anti-GFAP antibodies, illustrating the absence of inter-hemispheric differences in immunostaining levels.

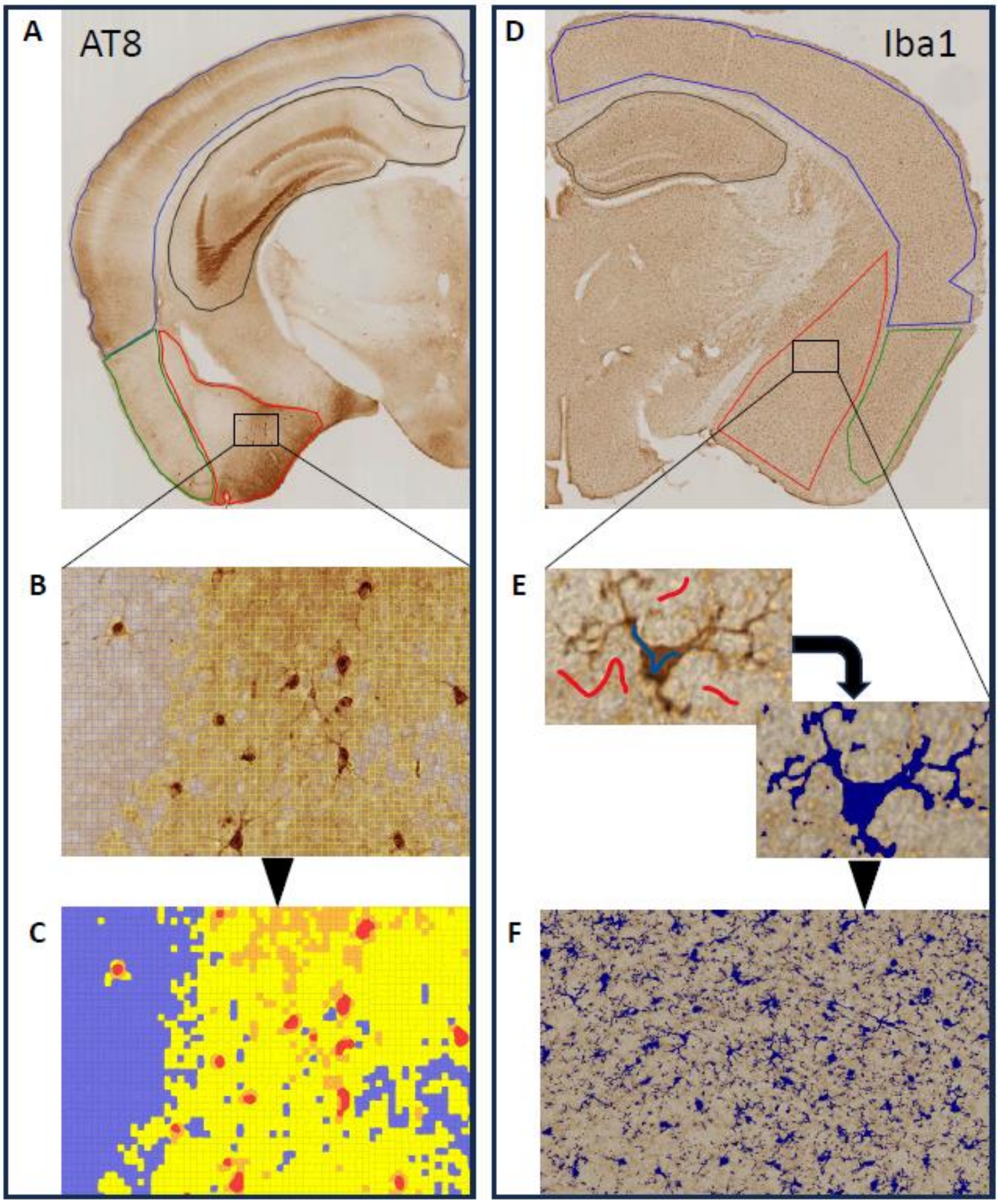


**Figure S2.** Image analysis for quantification of AT8 and Iba1 staining. A: Coronal section of a brain hemisphere immunostained with AT8 antibody. B: Superpixel segmentation of a zoomed region of the amygdala showing how superpixels are able to adapt their shape to local contrast variations. C: Visualization of the intensity map with negative (blue), weak (yellow), moderate (orange), strong (red) superpixels. D: Coronal section of a brain hemisphere immunostained with anti-Iba1 antibody. E: Representative images of manual annotations of positive signal (in blue) and background (in red). These annotations were used to train the machine-learning algorithm to detect iba1 staining (blue overlay in the right photo). F: Visualization of a zoomed region of the amygdala with positive signal determined by the machine-learning algorithm (microglia overlayed in blue).

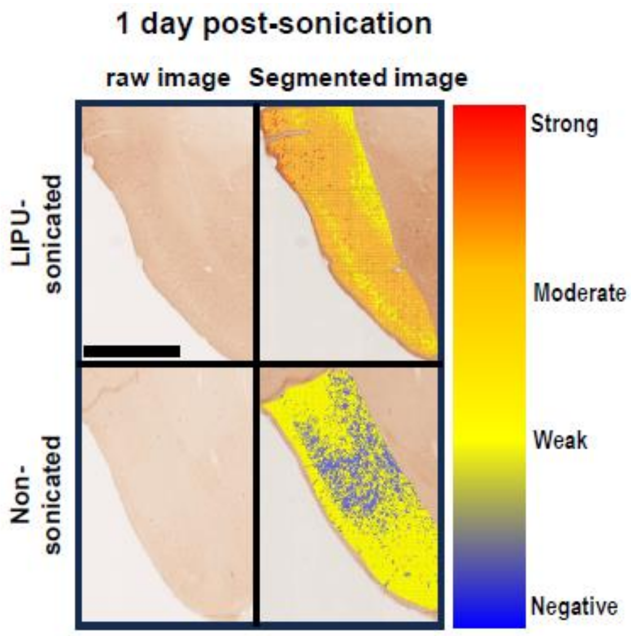


**Figure S3.** Tau pathology in raw images in the piriform cortex of a LIPU-sonicated mouse (upper panels) and a non-sonicated mouse (lower panels), (scale bar = 1 mm). Both mice were euthanatized one day after the last sonication session. An overall increase in signal intensities and distribution in the LIPU-sonicated brain can be observed.

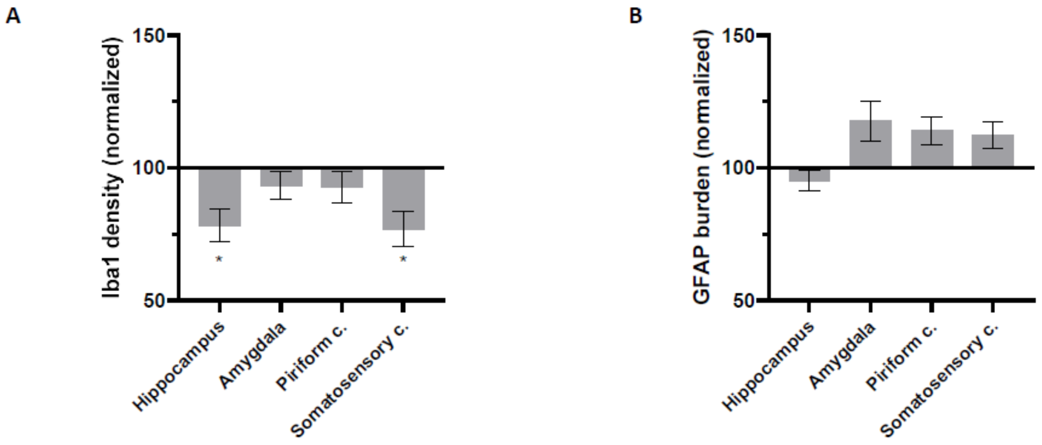


**Figure S4.** Effects of LIPU on glial cells (complementary analysis). A: Densities of microglial cells of LIPU-sonicated P301S mice (n=10) normalized to densities of non-sonicated P301S mice (n=7). LIPU sonication induced a decrease of microglial densities in the hippocampus and somatosensory cortex. B: GFAP loads of LIPU-sonicated P301S mice (n=10) normalized to loads of non-sonicated P301S mice (n=7). Variations in astrocytes burden induced by sonication did not reach statistical significance.
